# Physical interventions for people with more advanced dementia – a scoping review

**DOI:** 10.1186/s12877-021-02577-0

**Published:** 2021-12-04

**Authors:** Abigail J. Hall, Samantha Febrey, Victoria A. Goodwin

**Affiliations:** University of Exeter, Heavitree Road, Exeter, EX1 2LU UK

**Keywords:** Dementia, Rehabilitation, Physiotherapy, Exercise, Functional

## Abstract

**Background:**

Dementia is a neuro-degenerative condition resulting in cognitive and physical decline over time. In the early stages of the condition, physical decline may be slow, but in the later stages, it may become more pronounced. Physical interventions may be employed to try and reduce the physical decline that people experience, yet it is unclear what interventions may be effective. The aim of this study was to explore the breadth and quantity of evidence that exists in relation to the delivery of physical interventions for people with advanced dementia.

**Methods:**

We undertook a scoping review in order to map the current literature. All types of study design were included in the search in order to gain a comprehensive scope of the literature. We searched a variety of databases from inception until March 2021, focusing on physical interventions. Double screening and data extraction were employed in order to increase the reliability of the results.

**Results:**

Our review found four studies which focused on physical interventions aimed at improving physical outcomes for people with more advanced dementia. The majority of studies were excluded as their interventions were not specific to people with advanced dementia. The studies that were included incorporated functional activities and, despite small sample sizes, suggested statistically significant improvements in outcomes for people with advanced dementia.

**Conclusion:**

There is currently limited evidence relating to physical rehabilitation interventions for people with more advanced dementia, however, the evidence we presented suggests potential benefits for physical outcomes. Future research should focus on robust research to determine the most effective and cost-effective interventions that meet the needs of this population.

## Background

It is recognized that the global population is ageing and with people living longer [1] health and care costs are likely to increase. The total annual cost of dementia in England was estimated to be £24.2 billion in 2015, of which 42% (£10.1 billion) was attributable to unpaid care. Social care costs (£10.2 billion) were suggested to be three times larger than health care costs (£3.8 billion) [2]. Gait disturbances and impaired balance are common in people with dementia [3], significantly increasing the risk of falls [4]. Consequently, lower limb fractures are common in this population [3].

In the UK, it is estimated that there are 676,000 people living with dementia, with a predicted economic cost of approximately £26 million per year [5]. The increase in prevalence of dementia is reflected globally with the World Health Organization declaring dementia to be a public health priority [6] citing rapidly increasing global prevalence with figures rising from 36.5 million people living with dementia (PLwD) in 2010 to 115.4 million in 2050. With the increased numbers of PLwD, it is proposed that there will be an increased burden on caregivers, community and residential care services [7] as well as increased pressure and demand on healthcare systems.

Dementia is a term used to describe a set of disorders affecting the brain, which results in a global and continuing loss of cognitive and intellectual functioning, leading to difficulty maintaining social and occupational performance [8]. As a chronic and progressive disease, it in ultimately a fatal neurodegenerative disease [9]. While there are over 100 established different types of dementia [10], it can be broadly categorised into four main types: Alzheimer’s, vascular, Lewy Body and Frontotemporal – although many have mixed aetiologies. While the initial stages of dementia may only present with discrete and almost undetectable symptoms, advanced dementia is characterized by profound cognitive impairment, absence of verbal communication and complete functional dependence [9]. There are many tools used to attempt to define a person’s stage of dementia, with arguably the most common and highly validated being the Clinical Dementia Rating (CDR) [11], however there is considerable heterogeneity in scales that are used.

Several systematic reviews suggest the benefits of exercise in people with dementia to improve/maintain functional ability [12, 13], balance [14, 15], strength [15–17], mobility [12] and fitness [18]. Several other reviews of physical interventions have suggested improved cognitive function [13, 19–23] as well as improvements in levels of depression and behavioural difficulties [24] and ability to perform activities of daily living [22]. However, despite several authors reporting positive outcomes of exercise on cognition, another study [25], suggested that moderate to high intensity aerobic exercise and strength training had no effect on cognition, although there was a noted improvement in physical ability. A limitation of much of this evidence is the exclusion of people with more advanced dementia.

Therefore the aims of this study were to explore and map what evidence exists regarding physical rehabilitation interventions designed specifically for people with advanced dementia. We sought to identify gaps in the current evidence base to determine where further research should be focused [26].

## Methods

We used the methodologically rigorous scoping review approach in order to map the existing literature relating to rehabilitation interventions for people with advanced dementia, in terms of the volume, nature, and characteristics of the primary research [27]. Scoping reviews have been described as a form of comprehensive knowledge synthesis with the aim of informing practice and policy, while also providing direction to research priorities [27]. Initial exploratory literature searches demonstrated a paucity of literature relating to these aims, therefore it was hypothesised that there would be insufficient evidence to warrant undertaking a full systematic review. A scoping review methodology [27], without quality assessment [28], was adopted as the review sought to determine what evidence there was available initially. The protocol was registered with Open Science Framework (https://osf.io/nqmt8/ registered 18/11/2020).

In order to formulate a search strategy the PICO(S) method was employed [29] and the following inclusion and exclusion criteria were applied.

### Inclusion criteria

- People with any form of dementia reported as “severe/advanced” according to at least one of the following classifications MMSE < 20/30 or ACE-III < 64/100 or MOCA < 10/30
- Intervention included physical rehabilitation [30] or exercise provided on an individual basis, in group settings, at home, as an outpatient, in respite, nursing/care home settings, or in an in-patient setting. (The World Health Organisation define rehabilitation as “*a set of measures that assist individuals, who experience or are likely to experience disability, to achieve and maintain optimum functioning in interaction with their environments*” [6])
- Outcomes related to impairment, disability, participation, health related quality of life
- any study designs were considered including qualitative and quantitative studies. Only full papers were considered – abstracts or protocols were not included

### Exclusion criteria

- articles reporting on people with mild to moderate dementia or studies where the severity of the dementia is not reported

A comprehensive search of the literature was undertaken and the following databases were searched for articles from inception to search date (3rd January 2021); TRIP database, Cochrane Library (including ALOIS), Embase, Amed, PsycINFO, CINAHL, Medline (via Ovid), and PEDro.

Research registers (UK Clinical Trials Gateway and ISRCTN) were searched as well as PROSPERO to determine if there were any relevant trials or systematic reviews currently being undertaken. In order to gain a broad understanding of the literature, both positive and negative, grey literature searching took place using “Open Grey” and “ProQuest”. Keywords focused on dementia and terms related to rehabilitation or exercise (keywords 1-6 in the search strategy). Search terms around the types of study or outcomes were not used to prevent limiting the search. No authors were contacted specifically and no time limits were applied to the searches.

The search strategy was initially created in Medline (via Ovid) and then translated into the other databases. The following search strategy was employed (Table 1):

**Table 1:** Medline (via Ovid) search strategy

P	I
1. dement*.ti,ab,kw. 2. (cognitiv* adj declin*).ti,ab,kw. 3. (cognitiv* adj2 impair*).ti,ab,kw. 4. alzheimers.ti,ab,kw. 5. lewy body.ti,ab,kw 6. (chronic adj2 cerebrovas*).ti,ab,kw ***AND***	17. physiotherap*.ti,ab,kw. 18. (physic* adj2 therap*).ti,ab,kw. 19. rehabilitat*.ti,ab,kw. 20. exercis*.ti,ab,kw. 21. strength*.ti,ab,kw. 22. balance*.ti,ab,kw. 23. mobil*.ti,ab,kw. 24. (function* adj2 rehab*).ti,ab,kw. 25. exercise therap*.ti,ab,kw. 26. ((Therap* or train* or stimulat* or treatment* or program* or task*) adj2 (fit* or activit* or function* or recover*)).ti,ab,kw. 27. ((activit* or function*) adj2 recover*).ti,ab,kw. 28. ((Therap* or train* or stimulat* or activit* or function* or treatment* or program*) adj2 (Physical* or endurance or balance or strength* or flexibility or resistance or occupational or mobili*)).ti,ab,kw.
7. advanced.ti,ab,kw. 8. severe.ti,ab,kw. 9. chronic.ti,ab,kw. 10. end stage.ti,ab,kw. 11. institutional*.ti,ab,kw. 12. palliati*.ti,ab,kw. 13. long term care.ti,ab,kw. 14. nurs* hom*.ti,ab,kw. 15. (care adj2 home*).ti,ab,kw. 16. (late* adj2 stage*).ti,ab,kw.	
**29. 1 or 2 or 3 or 4 or 5 or 6** **30. 17 or 18 or 19 or 20 or 21 or 22 or 23 or 24 or 25 or 26 or 27 or 28** **31. 7 or 8 or 9 or 10 or 11 or 12 or 13 or 14 or 15 or 16** **32. 29 and 31** **33. 30 and 32**	

Following completion of all database searches, the citations were compiled and entered into EndNote bibliographic manager – where any duplicated citations were removed. Titles and abstracts were independently screened by two reviewers (AJH and SF). Discrepancies were discussed and consensus was gained by both reviewers prior to moving onto full text screening. Full text screening was then undertaken following the same process. Any disputes were discussed and consensus reached between reviewers. Should resolution of disputes not have been achieved, a third expert reviewer (VG) would have been consulted. Following full text screening, hand searching of the included studies was undertaken. This was conducted by analysing the bibliography of references for each study (backwards citation chasing) and through Google Scholar (forward citation chasing) and was necessary to ensure comprehensiveness of the search.

A standardised form was used to extract data from included studies. The form was piloted by the research team prior to using for full data extraction with two of the included studies. Both reviewers trialled the form and then discussed alterations that were needed. Once agreed, the new data extraction form was used for all included studies. Extracted information included: study population demographics, baseline characteristics, details of the experimental and control interventions, description of outcome measures and outcomes and was guided by the TIDieR checklist [31].

A narrative synthesis was undertaken to describe the articles included in terms of the type of study, participant characteristics, a summary of the intervention and tailoring of the intervention. This sought to describe the evidence available and identify the gaps in the current literature base.

## Results

**Fig. 1:**
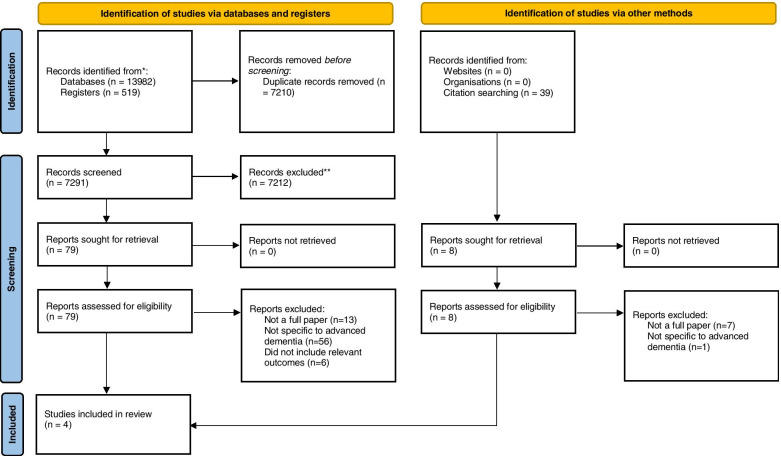
PRISMA diagram demonstrating the selection of articles for the scoping review. For more information, visit: http://www.prisma-statement.org/ [32]

From an initial database yield of 7240 articles, 79 full texts were reviewed following application of the eligibility criteria to the titles and abstracts (Fig. 1). Further application of the eligibility criteria to the full texts resulted in four selected papers for inclusion in the review. The main reasons for exclusion at full text screening was the failure of the intervention to be specific for people with advanced dementia (*n* = 56).

### Study and participant characteristics

All four of the included studies were randomised controlled trials (RCTs), with three being single centered [33–35] and Burge et al. being a multi-centre RCT [36]. The single centered trials were generally small with between 5 and 19 participants allocated to each group. Burge [36] included a larger number of participants with 136 allocated to the treatment arm and 134 to the control arm of the trial. See Table 2 for further details of included studies.

**Table 2:** Characteristics of included studies

	Burge [34]	Francese [30]	Kim [31]	Venturelli [33]
**Year**	**2017**	**1997**	**2016**	**2011**
**Country**	Switzerland	USA	Korea	Italy
**Design**	Multi-centre RCT	RCT	RCT	RCT
**Intervention group (N)**	136	6	19	12
**Control group (N)**	134	5	19	12
**Age**	81.7 (7.7)	Not reported	81.9 ± 7.0	83 (6)
**Intervention group (mean SD)**				
**Age**	81.1 (7.7)	Not reported	80.9 ± 6.1	85 (5)
**Control group (mean SD)**				
**Sex**	48.7	Not reported	13 (68.4)	Not reported
**Intervention group (N female,%)**				
**Sex**	53.7	Not reported	12 (85.7)	Not reported
**Control group (N female,%)**				
**Severity of dementia**	CDR ≥ 2	“Late stage”	Moderate to severe	MMSE 5 - 15
**Type(s) of dementia**	Alzheimer’s Lewy body Fronto-temporal Vascular Subcortical Mixed	Alzheimer’s	Alzheimer’s	Alzheimer’s
**Measurement of dementia**	CDR ≥ 2 (moderate to severe dementia), MMSE	Not reported	MMSE	MMSE
**Measurement of physical ability**	Barthel, FIM	Physical therapy assessment, Tinnetti	Exercise time, pedal rotation, total load, grip strength, Berg Balance Scale	6mwt, Barthel Index, glycaemia
**Intervention**	Usual care + exercise programme	Physical exercise programme	Physical exercise programme plus multicomponent cognitive programme	Walking
**Control**	Social interaction	Singing	Multicomponent cognitive programme	Daily organised activities
**Materials**	Demonstration by a therapist, music	Music, canes (for hand grips), bean bags, balls, weights + snack after 20 mins	TERASUERUGO - cycle ergometer	Walking log book
**Procedures**	Strengthening, balance and walking accompanied by music	Exercise 20 mins each morning 3/wk. for 7 wks	15 min of warm-up and stretching, 30 min of lower-limb aerobic exercise using a TERASUERUGO®, and 15 min of cool-down and relaxation.	Walking
**Who delivered**	Physical therapists, occupational therapists, or psychomotor therapists	Primary investigator plus volunteer(s)	Physical therapist	Physiotherapist and caregiver
**Method of delivery**	Groups of 4	Small groups	Not reported	Individual
**Location of intervention**	Acute psychiatric ward patients, but delivered off the ward	Dementia nursing facility	Long term care facility	Alzheimer’s care unit
**When/how much**	20 physical exercise sessions over 4 weeks lasting 30 mins each of moderate intensity	20 min 3x week for 7 weeks	60 min of supervised exercise sessions 5 times a week for 6 months	30 min of moderate exercise (walking) 4 times a week; the program consisted of a simple aerobic walking activity. 24 weeks
**Tailoring**	Individualised and graduated exercise programme	not reported	not reported	encouraged to walk at “fastest pace”
**Modifications**	none reported	Not reported	not reported	not reported
**Check of fidelity**	adherence	not reported	not reported	log books checked before and after walking session
**Actual fidelity**	81 completed out of 136 (most left hospital). 13.18/20 sessions	Not reported	not reported	93.4% + 3.2% presence at the 96 scheduled training session. 1 person in walking group left due to stroke
**Outcomes measured**	Effect on ADL	Depression, balance and physical ability (muscle strength, balance, ADL)	Cognition, grip strength, pedal power, total load, balance	Walking, ADL, cognition
**Outcome measures used**	Barthel index, FIM	CADS, physical therapy score (PT muscle strength test), Tinnetti	MMSE, Berg scale, Borg scale, Grip strength	6WT, BI, MMSE, glycaemia
**Results**	No significant differences. ADL scores deteriorate during acute psychiatric hospitalization. An exercise program delays the loss of mobility but does not have a significant impact on overall ADL scores.	Significant difference between groups for Tinetti measurement in favour of intervention.	Exercise time, pedal rotation, total load, grip strength and BBS was significantly increased at 6 months	Significant difference between groups for 6WT and BI in favour of intervention.
**Long term follow-up**	2 week follow-up	None	1,3 and 6 months	6 months

RCT - randomised controlled trial, CDR – Clinical Dementia Rating Scale, MMSE – Mini-mental state examination, FIM – functional independence measure, 6mwt – 6 min walking test, BI – Barthel Index

Where reported, the average age of the intervention groups were largely similar with the mean age of the intervention group being 81.7 to 83 years, reflecting the typical age group that would likely be living with advanced dementia. Only Kim and Burge reported the sex of their participants [34, 36] with 48.7 and 68.4% respectively being female in the intervention groups and 53.7 and 85.7% being female in the control groups.

Three of the interventions were delivered to residents in long term care facilities [33–35] whereas Burge et al. delivered the intervention to participants who were patients on an acute psychiatric ward [36]. Patients experiencing an acute illness may have affected their engagement in the intervention – indeed only 81 participants out of 136 recruited actually completed the intervention.

Severity of dementia was recorded and measured differently in the trials, but all included participants with more advanced dementia as their entire population. Measurements included the Clinical Dementia Rating Scale (CDR) Burge [36] and the Mini Mental State Examination (MMSE) [34–36]. Francese [33], failed to document a clear measure of dementia simply stating that it was documented in the medical notes that the participants had severe dementia.

There was significant heterogeneity in the physical outcome measures used in the studies with little consistency in outcome measures used. The Barthel Index was the only outcome measure used by more than one study [35, 36] and was one of only two functional outcome measures used, with Burge et al. (2017) also utilizing the Functional Independence Measure (FIM). The majority of the other outcome measures related to balance – the Berg Balance Scale [34] and the Tinetti balance assessment [33] or physical abilities such as walking with the use of the 6 min walking test [35] or grip strength [34]. Only Venturelli used a more biomedical outcome measure, measuring the level of glycaemia of the participant [35].

### Intervention

All studies sought to determine the efficacy of an exercise intervention with only Venturelli focusing solely on walking [35], while the others incorporated different types of strengthening, balance and cardiovascular components to their intervention. The studies described the actual intervention that was delivered (including components and materials required), who delivered it and where it was delivered comprehensively (Table 2).

Venturelli included a walking programme [35] of moderate intensity for 30 min, four times per week for a period of 24 weeks. The use of a cycle ergometer was explored by Kim et al. [34]. Their intervention, consisted of 15 min of warm-up and stretching, 30 min of lower-limb aerobic exercise using the cycle ergometer, and 15 min of cool-down and relaxation. The intensity of exercise was a heart rate of 40–60% of the maximum (Borg scale scores of 11– 13) and the dose consisted of 60 min of exercise repeated 5 days a week for 6 months. Alongside the control group, they also received a multi-component cognitive programme consisting of music therapy, art therapy, horticulture therapy, handicraft, recreational therapy, stretching, laughing therapy and activity therapy.

The remaining two studies incorporated a multi-component exercise intervention including strengthening and balance exercises. Francese and colleagues [33] delivered an intervention with a variety of basic exercises including balls and weights in small group exercise sessions that were delivered three times a week for 20 min every morning for 7 weeks to residents of a nursing facility for people with dementia. The other study incorporated strength, flexibility, walking, and balance training [36] and was based on an exercise intervention previously described by Rolland et al. [37]. This intervention focused on balance, lower-limb strengthening, flexibility and aerobic exercises.

### Tailoring intervention for dementia

None of the included studies reported any details regarding any tailoring or modification during the intervention with only one study suggesting that the intervention was tailored and graded according to the participant [36]. None of the studies include measures of fidelity to determine how accurately the intervention was delivered compared with the protocol.

### Study outcomes

Despite significant heterogeneity in the outcome measures used by the authors, the majority of studies reported improvements in physical function for the intervention groups compared to the control groups. Walking distance was reported to be significantly improved in the intervention group who received a walking exercise programme in the study by Venturelli [35]. This study reported that participants had a significant improvement in their 6 min walking test distance (294 m ± 49 for the walking group compared to 168 m ± 34 for the control group, *p* < 0.05) compared to a decline for the control group as well as a significant improvement in ability to undertake activities of daily living – according to the Barthel index (42 ± 4 post intervention in the walking group compared to 32 ± 4 in the control group, *P* < 0.05). There was a statistically significant difference in the MMSE score in the walking group compared to the control group (12 ± 2 compared to 6 ± 2, *p* < 0.05), with the authors concluding that as well as physical improvements, the walking programme has the potential to stabilize the progressive cognitive decline in nursing home residents.

Walking ability wasn’t measured in any of the other studies.

The use of a cycle ergometer was explored by Kim [34]. They reported positive outcomes for all of their measures including statistically significant increases after 6 months of, exercise time (207.7 ± 183.3 at baseline compared to 656.8 ± 315.5, *p* < 0.0001), number of pedal rotations (97.7 ± 89.9 at baseline compared to 285.8 ± 197.5, *p* < 0.004), total load (6.3 ± 7.5 at baseline compared to 10.0 ± 6.8, *p* < 0.06), grip (7.9 ± 5.9 at baseline compared to 11.87 ± 7.7p < 0.02) as well as a significant increase in Berg Balance Score (28.2 ± 17.6 at baseline compared to 21.5 ± 17.3, *p* < 0.04). However, they failed to report the physical outcome data for the control group, so between group differences were not presented.

Balance was reported in by Francese and Kim [33, 34] with the authors reporting statistically significant improvements for the intervention group in Berg or Tinetti scores. Francese and colleagues [33] described a significant difference between groups for Tinetti measurement in favour of the intervention (8.76 ± 4.32 compared to 0.4 ± 0.89, *p* < 0.05) and on the Physical Therapy Assessment (89.67 ± 10.03 compared to 43.60 ± 37.67 in the control group, *p* = 0.01). However, Kim et al. [34] failed to report the physical outcome data for the control group, so between group differences were not comparable. Their results showed no significant differences between the intervention and control group, suggesting minimal benefits of the exercise intervention.

Unlike the other studies which took place in care home facilities, Burge et al. recruited participants who were admitted to acute psychiatric units [36]. Their results showed no significant differences between the intervention and control group, suggesting minimal benefits of the exercise intervention, potentially due to the small sample size meaning that the study failed to allow enough power to detect differences between the intervention and control arms. Measures of ability to undertake activities of daily living were explored in three of the studies [33, 35, 36], Venturelli [35] reporting a difference in the Barthel index (42 ± 4 post intervention in the walking group compared to 32 ± 4 in the control group, *P* < 0.05). While it is not within the aims of a scoping review to undertake a quality assessment of included studies [28], it must be considered that the small sample sizes may cast doubt on the reliability of the results.

## Discussion

The aim of this study was to explore the breath and quantity of evidence that exists in relation to the delivery of physical interventions for people with advanced dementia. Our review is the first which has focused specifically on studies involving participants with more advanced dementia, with other studies tending to target people with mild to moderate dementia or deliver a single intervention designed to target all severities. The results suggest that there is a paucity of evidence relating to the efficacy of physical interventions designed specifically for people with more advanced dementia. Our review included just four studies which specifically delivered an intervention targeted at people with advanced dementia and included a range of physical interventions aimed at increasing activity levels. The majority of these studies suggested the positive effect of exercise for people with advanced dementia. There were no qualitative studies relating to physical rehabilitation for people with advanced dementia.

Intrinsic falls risk factors for people with dementia, such as impaired coordination, abnormalities of gait and postural instability progressively worsen throughout the disease process [38]. As the severity of dementia progresses, the decline in gait accelerates [39], with a concurrent increase in the risk of falls. It has been reported that the risk of falling is eightfold higher in people with dementia than in those without [40]. Therefore, understanding the interventions which may be effective at counteracting the physical declines people with advanced dementia experience could be important to reduce the negative consequences.

A previous systematic review, which included 1378 participants with all severities of dementia, suggested that intensive physical rehabilitation could improve mobility and had long term benefits in physical functioning [12]. A recent large RCT including participants with mild to moderate dementia suggested that there is limited evidence of the effectiveness of moderate to high intensity aerobic and strength exercise training programme on slowing cognitive function, but did improve physical fitness in the short-term [25]. This may relate to the type and method of delivery of the exercise, with the majority of the studies included in the systematic review incorporating functional exercises such as walking and dancing [12], in comparison to the more recent study which during supervised sessions used exercises such as static cycling and strength training using weights [25]. It could be hypothesized that the more functional approach to exercise is more beneficial to people with more advanced dementia.

Physiotherapists themselves have reported the challenges of delivering interventions for people with more advanced dementia [41, 42] and therefore future research should focus on interventions that are designed specifically for people with more severe dementia rather than applying a “one-size fits all” approach to interventions for people with dementia. Interestingly, none of the papers we included reported tailoring the intervention specifically for the participant. There is currently insufficient evidence to guide physical interventions for this population, therefore, high quality RCTs need to be undertaken in order to advise clinical practice for this population. It must also be considered that outcome measures need to reflect the population under study. The use of complex outcome measures - such as Berg Balance, as used in one of these studies, may not be appropriate for this population due to the cognitive capacity needed to undertake them. Therefore outcome measures should consider physical functioning or ability rather than biomedical measures of outcome.

### Strengths and limitations

For this study, a scoping review methodology was chosen in order to map the available evidence using a robust structure. Double screening was undertaken at all stages of the selection of articles in order to increase the reliability of the results and followed the methodology described by Arksey and O’Malley [27] and reporting of the results was guided by the TIDieR guidelines [31]. The quality of the included studies was not explored, as this is not within the remit (or the aims) of this type of study.

## Conclusion

There is currently minimal evidence to support physical interventions for people with more advanced dementia, however, the evidence we presented suggests potential benefits for physical outcomes. However, future research should focus on robust research to determine the most effective and cost-effective interventions that meet the needs of this population.

### Future recommendations

This study demonstrated a small amount of literature to support physical interventions for older people with more advanced dementia, however, the studies were small and therefore the results may not be reliable when translated to a larger population. Therefore, we suggest that physical interventions for this population need to be evaluated with larger populations in further RCTs.

## Data Availability

The datasets used and/or analysed during the current study available from the corresponding author on reasonable request.

## Abbreviations

6mwt: 6 min walking test
ACE: Addenbrooke’s Cognitive Examination
CDR: Clinical Dementia Rating Scale
FIM: functional independence measure
MMSE: Mini-mental state examination
MOCA: Montreal Cognitive Assessment
PLwD: People living with dementia
RCT: randomised controlled trial
